# ATM/ATR Phosphorylation of CtIP on Its Conserved Sae2-like Domain Is Required for Genotoxin-Induced DNA Resection but Dispensable for Animal Development

**DOI:** 10.3390/cells12232762

**Published:** 2023-12-04

**Authors:** Foon Wu-Baer, Madeline Wong, Lydia Tschoe, Chyuan-Sheng Lin, Wenxia Jiang, Shan Zha, Richard Baer

**Affiliations:** 1 Institute for Cancer Genetics, Columbia University Irving Medical Center, New York, NY 10032, USA; fw110@columbia.edu (F.W.-B.); mwong86@uw.edu (M.W.); lydiatschoe@gmail.com (L.T.); jw759@cumc.columbia.edu (W.J.); sz2296@cumc.columbia.edu (S.Z.); 2 Department of Pathology & Cell Biology, Columbia University Irving Medical Center, New York, NY 10032, USA; csl5@cumc.columbia.edu; 3 Herbert Irving Comprehensive Cancer Center, Columbia University Irving Medical Center, New York, NY 10032, USA; 4 Department of Pediatrics, Columbia University Irving Medical Center, New York, NY 10032, USA

**Keywords:** DNA resection, DNA break repair, homologous recombination, CtIP, Mre11, ATM/ATR phosphorylation, genotoxic stress

## Abstract

Homology-directed repair (HDR) of double-strand DNA breaks (DSBs) is dependent on enzymatic resection of DNA ends by the Mre11/Rad50/Nbs1 complex. DNA resection is triggered by the CtIP/Sae2 protein, which allosterically promotes Mre11-mediated endonuclease DNA cleavage at a position internal to the DSB. Although the mechanics of resection, including the initial endonucleolytic step, are largely conserved in eucaryotes, CtIP and its functional counterpart in *Saccharomyces cerevisiae* (Sae2) share only a modest stretch of amino acid homology. Nonetheless, this stretch contains two highly conserved phosphorylation sites for cyclin-dependent kinases (T843 in mouse) and the damage-induced ATM/ATR kinases (T855 in mouse), both of which are required for DNA resection. To explore the function of ATM/ATR phosphorylation at Ctip-T855, we generated and analyzed mice expressing the Ctip-T855A mutant. Surprisingly, unlike Ctip-null mice and Ctip-T843A-expressing mice, both of which undergo embryonic lethality, homozygous *Ctip*^T855A/T855A^ mice develop normally. Nonetheless, they are hypersensitive to ionizing radiation, and *Ctip*^T855A/T855A^ mouse embryo fibroblasts from these mice display marked defects in DNA resection, chromosomal stability, and HDR-mediated repair of DSBs. Thus, although ATM/ATR phosphorylation of CtIP-T855 is not required for normal animal development, it enhances CtIP-mediated DNA resection in response to acute stress, such as genotoxin exposure.

## 1. Introduction

The CtIP protein was originally identified on the basis of its association with the CtBP transcriptional co-repressor [1] and the BRCA1 tumor suppressor [2,3]. However, its physiological functions remained obscure until CtIP was recognized as the mammalian ortholog of Sae2 [4], a factor that promotes end resection of DNA double-strand breaks (DSBs) in the budding yeast *Saccharomyces cerevisiae* [5]. In this capacity, CtIP facilitates the processing of DSB ends by MRN, a trimeric protein complex comprised of the Mre11 nuclease, Rad50, and NBS1 (Xrs1 in budding yeast) [4,6,7,8]. In eukaryotic cells, DSBs are repaired primarily through either of two pathways: canonical non-homologous end-joining (c-NHEJ) or homology-directed repair (HDR). While c-NHEJ is active throughout the cell cycle, HDR is largely restricted to the S and G2 phases, when sister chromatids are available to serve as templates for homologous recombination. DNA resection favors HDR by converting DSB ends into 3′-single-strand DNA (ssDNA) overhangs, which initiate ATR-dependent checkpoint signaling and, when assembled into a Rad51/ssDNA nucleofilament, can invade a homologous DNA duplex [6]. In addition to the c-NHEJ and HDR pathways, DSBs can also be repaired in some settings by alternative end ligation (alt-EJ), which entails the annealing of ssDNA microhomologies generated at both DNA ends by short-range resection. Although CtIP is required for DSB repair by alt-EJ in most cell types [9,10], it is dispensable for alt-EJ-mediated class switch recombination of the immunoglobulin heavy chain (IgM) gene during B cell development, presumably because the highly repetitive and GC-rich switch region sequences provide optimal substrates for resection-independent alt-EJ [11].

Given its central role in DSB repair choice, the molecular factors that initiate DNA end resection are of keen interest [6]. The Mre11 subunit of the MRN complex possesses both an endonuclease activity and a 3′-5′ exonuclease activity. Short-range DNA resection is triggered by Mre11-mediated endonucleolytic cleavage of the 5′-terminated DNA strand at a position internal to the DSB end [12,13,14]. The 3′-5′ exonuclease activity of Mre11 then digests the 5′-strand, moving from the internal nick toward the DSB end. The 3′-ssDNA overhang generated by this process can be further extended (long-range resection) by the Exo1 exonuclease or by the coordinated actions of the DNA2 endonuclease and a RecQ-family helicase [15,16]. Importantly, the “clipping” reaction that initiates short-range resection is promoted by CtIP^Sae2^, which acts as an allosteric co-factor for the endonuclease activity of Mre11 [6,7,12,13,14]. In addition, CtIP also facilitates long-range resection by promoting the recruitment of Exo1 to DSB ends [17] and stimulating both the exonuclease and helicase activities of the DNA2/BLM complex [18,19]. While yeast Sae2^CtIP^ is only required for the resection of blocked DNA ends, such as the Spo11-induced breaks generated during meiosis, mammalian CtIP^Sae2^ is also critical for MRN-mediated processing of unblocked ends [6]. As such, mammalian CtIP controls a pivotal decision point in the DNA damage response by initiating DSB repair through either the HDR or alt-EJ pathways. In doing so, it also shapes the timing and duration of cellular responses to DSBs. For example, CtIP^Sae2^-mediated resection both mitigates ATM^Tel1^ signaling by removing the MRN^MRX^ complex from DSB ends and activates ATR^Mec1^ signaling by promoting the formation of ssDNA/RPA filaments [20,21,22]. Given the integral functions of CtIP in DNA resection and cell cycle checkpoint control, it is not surprising that Ctip-null mice undergo early embryonic lethality, and in most settings, cell viability is lost in the absence of CtIP [7,23,24,25].

The relationship between human CtIP and yeast Sae2 was originally recognized on the basis of a short amino acid homology between the carboxy-terminal sequences of both proteins (Figure S1) [4,7]. CtIP/Sae2 orthologs that function in DNA resection and share this C-terminal Sae2-like homology domain have since been found throughout the eukaryotic kingdom [26,27,28]. A striking feature of the Sae2-like domain is the presence of two highly conserved phosphorylation sites, one of which serves as a substrate for cyclin-dependent kinases (CDKs) [29,30] and the other for certain phosphoinositide 3-kinase-related protein kinases (PI3KKs), such as ATR and ATM [17,31]. Phosphorylation of the CDK site (T847 in human CtIP) is essential for CtIP to promote the MRN clipping reaction that initiates DNA resection [13,29,30]. Mechanistically, T847-phosphorylated CtIP induces clipping by binding the FHA/BRCT domains of NBS1, which in turn allows NBS to elicit the endonuclease activity of Mre11 [32]. Likewise, phosphorylation of the corresponding residue of Sae2 (S267) also promotes clipping by the MRX complex in yeast [33]. Accordingly, mutations that ablate this site (e.g., CtIP-T847A or Sae2-S267A) cause genomic instability and severely impair resection-dependent cellular functions such as HDR and ATR-dependent checkpoint activation [29,30]. Interestingly, the viability of Ctip-null mice can be restored by ectopic expression of the wildtype but not the T847A-mutant human CtIP protein [34]. In light of this observation, it is possible that animal development is dependent on CDK-mediated CtIP-T847 phosphorylation and the MRN clipping reaction that it sets in motion.

The C-terminal Sae2-like homology domain of CtIP also possesses a highly conserved phosphorylation motif (T859 in human CtIP and T855 in mouse Ctip) recognized by the ATR and ATM kinases. In response to DNA damage, ATR/ATM-mediated phosphorylation of CtIP-T859 facilitates the stable association of CtIP with DSB-containing chromatin and promotes optimal DNA resection and HDR repair of DSBs [17,31]. Although ATR/ATM phosphorylation of this site also promotes DNA resection in B lymphocytes, it is not required for programmed immunoglobulin gene rearrangements such as V(D)J recombination and class switch recombination [25,35]. Here we show that mice bearing a missense mutation that eliminates this phosphorylation site (Ctip-T855A) develop normally but are hypersensitive to genotoxic stress. Moreover, cells from these mice display marked defects in genotoxin-induced DNA resection, HDR repair of DSBs, and maintenance of chromosome stability. These results indicate that ATR/ATM phosphorylation of Ctip-T855 enhances DNA resection in response to genotoxic stress, but unlike CDK phosphorylation of Ctip-T847 DNA, it is dispensable for normal animal development.

## 2. Materials and Methods

### 2.1. Generation of Ctip^T855A^ Mice

All mice were housed in an AAALAC-accredited facility at Columbia University Irving Medical Center and studied according to protocols approved by the Columbia University Institutional Animal Care and Use Committee. To generate mice harboring the *Ctip*^T855A^ allele, a knock-in targeting vector including *Ctip* exons 16–19 was constructed by inserting a neomycin-resistance gene cassette flanked by loxP sites (loxP-PGK-neo-loxP) into intron 18 and replacing the T855 codon (ACT) of exon 18 with a codon for alanine (GCT) (Figure S2a,b). KV1 embryonic stem (ES) cells [36] were then electroporated with the targeting vector. Properly recombined neomycin-resistant ES clones were identified by both Southern analysis and the presence of the desired T855A coding mutation confirmed by nucleotide sequence analysis of a 565 base-pair DNA fragment generated by PCR amplification with a forward Ctip-19 (TCAGTAAAATGCCACTCTGG) and reverse Ctip-O (TGCCCACTTTTGAAGGCACTGAGTC) oligonucleotide. Two independent clones of *Ctip*^T855A-neo/+^ ES cells were injected into C57BL/6J blastocysts for the production of germline-transformed mice. The loxP-PGK-neo-loxP cassette was then excised from the targeted allele (Figure S2c) by mating chimeric male *Ctip*^T855A-neo/+^ mice with females carrying a ubiquitously expressed *Cre* transgene (EIIa-Cre; B6.FVB-Tg(EIIa-cre)C5379Lmgd/J; strain 003724, (Jackson Laboratory, Bar Harbor, ME, USA). Offspring heterozygous for the desired *Ctip*^T855A^ allele (Figure S2d) were identified by PCR amplification of tail DNA with a forward Ctip-20 (5′-GCCTGGCTTGGCATTCAGATTTCA-3′) and reverse Ctip-T (5′-ATGCCATAGACCTCATAGTC-3′) oligonucleotide, which generates distinct DNA fragments for the wildtype *Ctip*^+^ (338 base pairs) and mutant *Ctip*^T855A^ (248 base pairs) alleles. The *Ctip*^T855A/+^ mice were then backcrossed twice with pure C57Bl/6J mice (Jackson Laboratory stock 000664) to yield animals that were approximately 94% C57BL/6J (N3 backcrossed), and the mouse embryonic fibroblast (MEF) and embryonic stem (ES) cell lines were generated using mice on this background. All bred mice and all derived cell lines were (1) genotyped by PCR amplification as described above to distinguish the *Ctip*^+^ and *Ctip*^T855A^ alleles and (2) subjected to nucleotide sequence analysis to confirm the presence of the T855A codon on the *Ctip*^T855A^ allele (Figure S2e). To evaluate IR sensitivity, five-week-old littermates of *Ctip*^+/+^, *Ctip*^T855A/+^, and *Ctip*^T855A/T855A^ mice were subjected to whole-body radiation (7.5 Gy) inside the turnable chamber of an MKI-30 SN 1172 Cesium-137 irradiator and then monitored for survival for 3 months. The *Ctip*^T855A^ mouse strain is available from Jackson Laboratory (strain # 037396).

### 2.2. Generation of Cell Lines

Mouse embryonic fibroblasts (MEFs) were derived from the progeny of a *Ctip*^T855A/+^ intercross by obtaining embryos from pregnant mothers at 13.5 days post-fertilization. Primary MEFs were cultured and immortalized with SV40 large T antigen as described [37], and the individual *Ctip*^+/+^, *Ctip*^T855A/+^, and *Ctip*^T855A/T855A^ MEF clones used in this study are listed in Table S1. The isogenic pairs of *Ctip*^+/+^ and *Ctip*^S326A/S326A^ MEFs were described previously [38]. Embryonic stem (ES) cells were derived from day E3.5 blastocysts and cultured as described previously [37]. Independent subclones of *Ctip*^+/+^ (A, B, and C) and *Ctip*^T855A/T855A^ (D, E, and F) ES cells possessing a DR-GFP recombination reporter integrated into the Pim1 locus were then generated using the p59xDR-GFP6 plasmid [39]. The isogenic pair of *Brca1*^+/+^ and *Brca1*^S1598F/S1598F^ ES subclones with a Pim1-integrated DR-GFP reporter were described previously [40].

### 2.3. Clonogenic Survival Assays

For cell survival assays, immortalized MEFs were seeded on 6-well plates at 1000 cells/well and evaluated in triplicates for each experimental condition, as described previously [37]. Thus, at 48 h after plating, cells were subjected to varying doses of ionizing radiation (0, 2, 4, 6, or 8 Gy) using an Atomic Energy of Canada Gammacell 40 Cesium unit or ultraviolet radiation (0, 0.34, 0.68, 1.36, 2.04, or 4.08 J/m^2^) using a UVGL-58 lamp (short-wave UVC calibrated with UVX-25 radiometer), or exposed to mitomycin C (0, 50, 100, 200, or 800 ng/mL) for 4 h, hydroxyurea (0, 0.06, 0.26, 1.02, or 4.10 µM) for five hours, neocarzinostatin (0, 25, 50, 75, 100, or 200 ng/mL) for one hour, camptothecin (0, 0.05, 0.10, 0.20, 0.40, or 1.00 µM) for one hour, or etoposide (0, 0.1, 0.3, 0.8, 2.0, or 5.0 µM) for one hour. Treated and mock-treated cells were then washed twice with PBS and cultured in fresh media. At 5–7 days post-treatment, the cells were stained with 0.2% crystal violet in a 50% methanol solution, and the surviving colonies (containing > 50 cells) were counted. For olaparib treatment, MEFs were exposed to varying concentrations (0, 0.064, 0.16, 0.4, 1.0, or 2.5 µM) at 24 h after plating, and the media were replaced every 48 h with fresh media containing the corresponding concentrations of olaparib until the time of cell harvest, 6–8 days after initial drug treatment.

### 2.4. Alkaline Comet Assays

To assess DNA damage using the alkaline comet assay, immortalized MEFs were seeded onto 12-well plates and treated the following day with either 10 Gy ionizing radiation (IR), 1 µM camptothecin (CPT) for one hour, 2 mM hydroxyurea (HU) for five hours, or 40 ng/mL mitomycin C (MMC) for 16 h. Immediately following genotoxic exposure, 4 × 10^5^ cells were mixed with 50 µL of 0.5% low-melting agarose/PBS, and the mixture was pipetted onto 2-well glass slides (Trivegen/Bio-Techne, Minneapolis, MN, USA). Once the agarose mixture had solidified, the slides were incubated with pH 10.0 lysis buffer (10 mM Tris-HCl, 2.5 M NaCl, 100 mM EDTA, and 1% Triton X-100) at 4 °C overnight while protected from light. The following day, the slides were equilibrated in pre-chilled (4 °C) electrophoresis buffer (300 mM NaOH, 1 mM EDTA) for 20 min. After electrophoresis in a horizontal chamber (Fisher Scientific, Waltham, MA, USA) for 40 min at 20 V (constant volts) at 300 mA, the slides were incubated twice in water for 5 min, fixed in 70% ethanol for 5 min, left to dry at 37 °C for 40 min, stained with SyBr Gold fluorescent dye (1:30,000 in 10 mM Tris-HCl pH 8.0, 0.1 mM EDTA) for 30 min, rinsed with water, and dried at room temperature. The stained comets were then imaged on an Eclipse 80i fluorescent microscope (Nikon, Meville, NY USA) with a CoolSNAP HQ2 camera (Telegyne Photometrics, Tucson, AZ, USA) at 10× magnification. Comet tail moment values were determined using CometScore Software Version 1.5. At least 75 tails were analyzed per experimental condition. Apoptotic cells (a small comet head and a very large comet tail) were excluded from the analysis.

### 2.5. T-FISH Assays

To assess chromosomal abnormalities by telomere fluorescent in situ hybridization (T-FISH), primary MEFS (passage 3 or earlier) were plated on 0.2% gelatin-coated plates and allowed to attach overnight. Upon reaching exponential growth at 48 h, the cells were treated (or mock-treated) with either 40 ng/mL MMC for 16 h or 1 µM CPT for 1 h. After drug exposure, Karyomax Colcemid (Thermo Fisher Scientific, Waltham, MA, USA) was added to each plate to a final concentration of 100 ng/mL, and the cells were incubated for an additional 4 h. The cells were then harvested, treated in hypotonic buffer (0.03 M Sodium citrate) at 37 °C for 25 min, fixed in a methanol/acetic acid (3:1) solution, and dropped onto glass microscope slides to obtain metaphase spreads. The telomeres were then stained with the Cy3-PNA probe (PNA Bio, Thousand Oaks, CA, USA), and the DNA was counterstained with DAPI-containing mounting media (Vectashield; Vector Laboratories, Newark, CA, USA). The T-FISH metaphase spreads were imaged on an Axio Imager Z2 fluorescent microscope with Coolcube1 camera (Zeiss, Hebron, KY, USA), and Metafer software version 3.10.6 (Metasystems, Newton, MA, USA) was used to automatically locate metaphases at 10× magnification and automatically capture images at 63× magnification (Cytogenetics Shared Resource, HICCC). The captured metaphases were then analyzed using Isis fluorescent imaging system software version 3.10.6 (Metasystems).

### 2.6. Western Blotting

For Western blotting, MEFs were seeded at 0.3 to 0.5 × 10^6^ cells per 100-mm plate. At 48 h, the exponentially growing cells were treated with 1 µM CPT for 1 h or for the time points 0, 20, 60, and 90 min. Treated and mock-treated cells were then harvested, lysed in 5× packed cell volumes of LS lysis buffer (10 mm Hepes, pH 7.6, 0.25 M NaCl, 0.1% NP40, 5 mM EDTA, 10% glycerol, EDTA-free complete proteases inhibitor cocktail (Roche, Indianapolis, IN, USA), 1 mM DTT, and 50 mM NaF) on ice for 10 min, and spun at maximum speed in a microcentrifuge for 10 min at 4 °C. The supernatants were then collected as cell lysates, and 50 μg protein aliquots of each lysate were fractionated by electrophoresis through 6.5% polyacrylamide gels in running buffer (25 mM Trizma base, 192 mM glycine, 0.1% SDS, pH 8.3) and electroblotted onto an Amersham Protran 0.45 µm nitrocellulose membrane (GE Healthcare Life Sciences, Chicago, IL, USA) in transfer buffer (25 mM Tris-HCl pH 7.6, 190 mM glycine, 20% methanol, 0.04% SDS) at 22 volts. After blocking with 10% milk in TBS-T buffer (20 mM Tris-HCl pH 7.6, 0.137 M NaCl, 0.1% Tween 20) for 5 min on a shaking platform, each membrane was incubated for 2 h at room temperature with one of the following primary antibodies in 2% milk/TBS-T: Brca1 rabbit polyclonal (1:2000) [40], Ctip 14-1 mouse monoclonal (1:50) [41], phospho-KAP-1 (S824) rabbit polyclonal (1:2000; Bethyl, Houston, TX, USA), KAP-1 rabbit polyclonal (1:20,000; Bethyl, Houston, TX, USA), phospho-Chk1 (S345) rabbit polyclonal (1:1000; Cell Signaling, Danvers, MA, USA), Chk1 (G4) mouse monoclonal (1:500; Santa Cruz, Dallas, TX, USA), phospho-RPA2 (S4/S8) rabbit polyclonal (1:2000; Bethyl, Houston, TX, USA), RPA2 rabbit polyclonal (1:20,000; Bethyl, Houston, TX, USA), and *α*-tubulin mouse monoclonal DM1A (1:10,000; Millipore, Burlington, MA USA). After incubation with the appropriate secondary antibody (GE Healthcare, Chicago, IL USA, NA934 Donkey anti-rabbit HRP at 1:10,000 dilution or Sigma, Burlington, MA USA, A5278 goat anti-mouse HRP at 1:10,000 dilution), each membrane was developed by chemiluminescence using either the SuperSignal West Pico substrate or the SuperSignal West Dura substrate (ThermoFischer Scientific, Waltham, MA USA).

### 2.7. Immunofluorescence Microscopy

For immunofluorescent microscopy of phospho-RPA2 and gH2AX foci, 10^5^ MEFs were seeded onto poly-l-lysine (Sigma)-coated coverslips. After 48 h, the cells were treated or mock-treated with 1 µM camptothecin (Sigma) for 1 h and then incubated on ice for 5 min with pre-extraction buffer (100 mM Pipes, pH 6.8, 2 mM EGTA, 1 mM MgCl_2_, 0.5% Triton X-100) and 3 min with strip buffer (100 mM Tris-HCl, pH 7.4, 10 mM NaCl, 3 mM MgCl_2_, 1% Tween 20, 0.25% sodium deoxycholate). After fixing with 4% paraformaldehyde (*w*/*v*) in PBS for 15 min, the cells were incubated in serum-free DMEM for 5 min, washed twice with PBS, and permeabilized with 1% Triton X-100 in net gel (50 mM Tris-HCl, pH 7.4, 150 mM NaCl, 5 mM EDTA, 0.05% NP40, 0.25% Gelatin IV (bloom 75, type B), 0.02% sodium azide) at 4 °C for 10 min. After washing twice with net gel, the coverslips were blocked for 15 min with 1% BSA in PBS. For immunofluorescent staining, the cells were first incubated with either phospho-RPA2 (T21) rabbit polyclonal antibody 1:100 (Abcam, Waltham, MA USA) or phospho-H2AX (Ser139) mouse monoclonal antibody 1:200 (Millipore, Burlington, MA USA) in a humidified chamber for 40 min, washed three times with 1% BSA/PBS, and then incubated for 40 min in 1:1000 dilutions of, respectively, goat anti-rabbit Alexa 488 or goat anti-mouse Alexa 568 (Molecular Probes, Waltham, MA USA). After staining, the coverslips were washed twice with 1% BSA/PBS, rinsed with PBS and then water, and mounted with Vectashield hard set mounting media containing 4′, 6-diamidino-2-phenylindole (DAPI; Vector Laboratories, Newark, CA, USA). The cells were imaged on a Nikon Ti Eclipse inverted confocal microscope with a Nikon A1 Plus camera at 60× magnification. For automated quantification of the pRPA2 and gH2AX foci, slides were stained with phospho-RPA2 (T21) rabbit polyclonal antibody 1:100 (Abcam Waltham, MA USA) or phospho-H2AX (S139) rabbit polyclonal antibody 1:200 (Cell Signaling, Danvers, MA, USA) as primary antibodies and incubated with 1:1000 diluted secondary antibodies of goat anti-rabbit Alexa 488 (Molecular Probes, Waltham, MA USA) as described above. Imaging was carried out using the Metafer software version 4 (Metasystems, Newton, MA USA), and 2000 cells were counted on each slide from three independent experiments.

### 2.8. DR-GFP and Single-Molecule Analysis of Resection Tracks (SMART) Assays

Double-strand DNA break repair by homologous recombination was evaluated in ES cell subclones harboring a single DR-GFP reporter integrated into the *Pim1* locus [39] as described previously [37]. For each experimental condition, 50,000 cells were analyzed by Flow Jo (version 10) software. For SMART assays [42], MEF cells were seeded on 1 × 100 mm plates at 0.25 to 0.3 × 10^6^ cells per plate. The next day, the cells were provided with fresh media containing 10 µM BrdU (Sigma) and cultured for 24 h. The cells were then either irradiated (10 Gy, Gammacell 40, cesium-137) and harvested 1 h later or treated with 1 µM CPT for 1 h. The cells were then embedded in low-melting agarose (Bio-Rad, Hercules, CA USA), followed by DNA extraction using the FiberPrep DNA extraction Kit (Genomic Vision, Bagneux, France). To stretch the DNA fibers, ComdiCoverslips (Genomic Vision) were dipped into the DNA solution for 15 min and pulled out at a constant speed (250 µm/s) using the FiberComb machine (Genomic Vision). Coverslips were baked for 2 h at 60 °C and incubated with anti-BrdU mouse monoclonal antibody 1:1000 (GE) in BlockAid (Molecular Probe) buffer for 1 h. The coverslips were washed three times in PBS with 0.05% Tween 20, incubated with anti-mouse Alexa 594 antibody 1:1000 in BlockAid buffer for 40 min, washed three times in PBS with 0.02% Tween 20, rinsed in water, and dehydrated in 70%, 90%, and 100% ethanol, respectively. The dried coverslips were mounted with Prolong Gold mounting media (Invitrogen) containing 1:15,000 YOYO1 (Invitrogen), and images of the DNA fibers were collected on an Eclipse 80i fluorescent microscope (Nikon) with a CoolSNAP HQ2 camera (Telegyne Photometrics, Tucson, AZ, USA) at 20× magnification and recorded using NIS ELEMENTS Nikon software version 4. DNA fiber lengths were measured by Fuji Image J, and a minimum of 230 fibers were analyzed for each sample.

### 2.9. Statistical Analyses

Statistical analyses were conducted on PRISM software version 6 (GraphPad), and significant differences were labeled with one, two, three, or four asterisks for *p* < 0.05, *p* < 0.01, *p* < 0.001, or *p* < 0.0001. The Mann–Whitney test was used to ascertain significant differences between the survival of distinct mouse cohorts, as well as the lengths of DNA fragments generated in the comet and SMART assays. The paired Student’s *t* test was used for all other statistical determinations.

## 3. Results

### 3.1. Mice Homozygous for the Ctip^T855A^ Allele Develop Normally, but Are Hypersensitive to Ionizing Radiation

Mammalian Ctip possesses two phosphorylation sites within its C-terminal Sae2-like homology domain that are stringently conserved across a broad phylogenetic spectrum: one serves as a substrate for CDK family kinases (T843 in mouse Ctip), while the other can be phosphorylated by PI3KK-like kinases, such as ATR and ATM (T855 in mouse Ctip) (Figure S1). Mice lacking the conserved CDK site undergo embryonic lethality [34], possibly due to a requirement for CDK-phosphorylated Ctip to induce Mre11 endonuclease activity and thereby initiate DNA resection [6]. To evaluate the role of Ctip-T855 phosphorylation in mammalian development, we introduced the T855A missense mutation into mouse ES cells by homologous recombination. The targeting vector contained genomic DNA encompassing exons 16-19 of the mouse *Ctip* gene, as well as the T855A mutation in exon 18 and a neomycin-resistance gene cassette flanked by *loxP* sites (loxP-neo-loxP) inserted into intron 18 (Figure S2a,b). Upon electroporation of ES cells with this construct, neomycin-resistant ES clones bearing the recombined *Ctip*^T855A-neo^ allele (Figure S2c) were identified and injected into blastocysts to produce germline-transformed mice. To excise the loxP-neo-loxP cassette from the targeted allele, chimeric male *Ctip*^T855A-neo/+^ mice were mated with females carrying a ubiquitously expressed *Cre* transgene (E2A-Cre) to produce offspring with the desired *Ctip*^T855A^ allele (Figure S2d).

**Figure 1:**
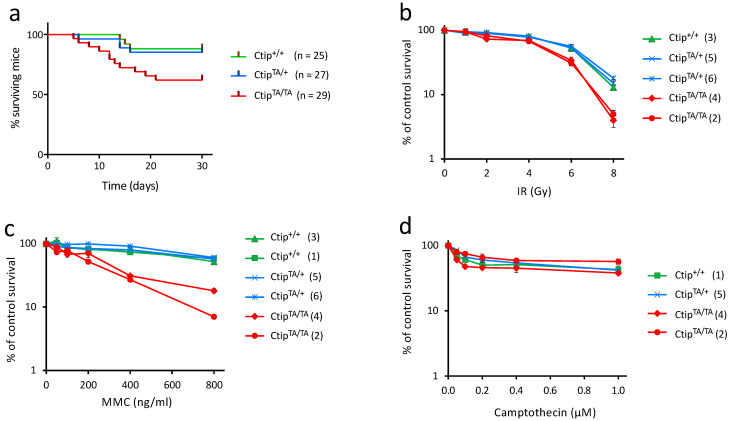
Homozygous *Ctip*^T855A/T855A^ mice and cells are hypersensitive to genotoxic stress. (**a**) Littermates of *Ctip*^+/+^, *Ctip*^T855A/+^, and *Ctip*^T855A/T855A^ mice were subjected to whole-body irradiation (7.5 Gy) at five weeks of age and monitored for survival over the next three months. The number of mice in each genetic cohort is indicated. As determined by the log-rank (Mantel-Cox) test, the survival of *Ctip*^T855A/T855A^ mice was significantly reduced relative to their C*tip*^T855A/+^ (*p* = 0.04) and *Ctip*^+/+^ (*p* = 0.027) littermates. In contrast, the survival of C*tip*^T855A/+^ mice was statistically indistinguishable from that of *Ctip*^+/+^ mice (*p* = 0.74). (**b**–**d**) Colony survival analysis of isogenic clones of *Ctip*^+/+^, *Ctip*^T855A/+^, and *Ctip*^T855A/T855A^ mouse embryonic fibroblasts (MEFs) exposed to ionizing radiation (**b**), mitomycin C (**c**), or the PARP inhibitor olaparib (**d**). Survival was quantified as the percentage of colonies on genotoxin-treated cultures relative to untreated cultures. Each condition was tested in triplicate, and error bars represent SEM. For each culture, the *Ctip* genotype is indicated, and the specific MEF clone analyzed is denoted by the number in parentheses as listed in Table S1.

Upon intercrossing heterozygous *Ctip*^T855A/+^ mice, we obtained heterozygous and homozygous mutant pups at the expected Mendelian ratios of ~50% and ~25%, respectively. The mutant mice developed normally and appeared indistinguishable from their wild-type littermates with respect to weight, fertility, and longevity. Thus, unlike the CDK phosphorylation site at Ctip-T843 [34], the ATR/ATM phosphorylation site at CtIP-T855 is not required for normal mouse development in the absence of acute stress. However, since the ATR/ATM kinases are enzymatically induced by DNA damage and act to coordinate cellular responses to that damage, we examined the sensitivity of adult mice to ionizing radiation. As shown in Figure 1a, whole body radiation (7.5 Gy) significantly reduced the survival of homozygous *Ctip*^T855A/T855A^ mice relative to both their heterozygous (C*tip*^T855A/+^) and wildtype (*Ctip*^+/+^) littermates.

### 3.2. Homozygous Ctip^T855A/T855A^ Cells Are Hypersensitive to a Subset of Genotoxic Agents

To examine the role of Ctip-T855 phosphorylation at the cellular level, heterozygous *Ctip*^T855A/+^ mice were intercrossed, and isogenic panels of mouse embryonic fibroblasts (MEFs) were derived from the wild-type, heterozygous-mutant, and homozygous-mutant embryos of three mothers (Table S1). As shown in Figure S3a, comparable expression of the Ctip polypeptide was observed in wild-type (*Ctip^+^*^/+^) and mutant (C*tip*^T855A/+^ and C*tip*^T855A/T855A^) cells. Of note, the electrophoretic mobility shift induced by genotoxic stress, such as camptothecin exposure, was not fully abrogated in C*tip*^T855A/T855A^ cells (Figure S3a), indicating that loss of T855 phosphorylation does not markedly compromise the modification of other DNA damage-induced phosphorylation sites within Ctip.

To determine whether T855 phosphorylation affects cellular responses to DNA damage, we used clonogenic survival assays to evaluate the sensitivity of Ctip-mutant MEFs to various genotoxic agents. In accordance with the effects of ionizing radiation (IR) on adult mice (Figure 1a), C*tip*^T855A/T855A^ MEFs displayed a modest, but consistent, hypersensitivity to IR relative to heterozygous and wild-type MEFs (Figure 1b). In addition, C*tip*^T855A/T855A^ cells are especially sensitive to the DNA crosslinking agent mitomycin C (MMC) (Figure 1c) and the PARP inhibitor olaparib (Figure S3b), but display little, if any, hypersensitivity to ultraviolet light, hydroxyurea, the radiomimetic neocarzinostatin (NCS), or the topoisomerase inhibitors camptothecin and etoposide (Figure 1b and Figure S3f).

### 3.3. Homozygous Ctip^T855A/T855A^ Cells Display Elevated DNA Damage in Response to Certain Genotoxic Agents

**Figure 2:**
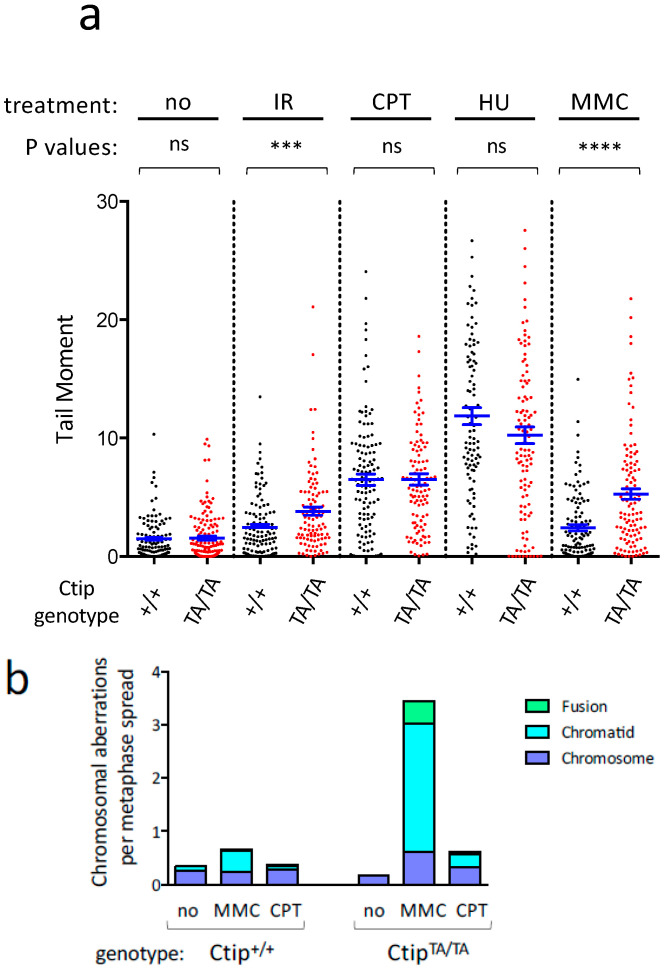

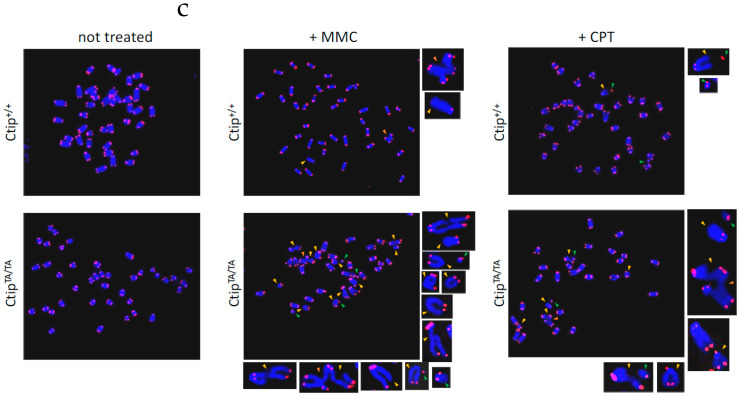
*Ctip*^T855A/T855A^ cells display elevated levels of DNA damage in response to specific genotoxic agents. (**a**) Levels of DNA damage in isogenic *Ctip*^T855A/T855A^ (clone 8) and Ctip^+/+^ (clone 7) MEFs were quantitated using the alkaline comet assay. Cells were either left untreated (no) or exposed to ionizing radiation (IR), camptothecin (CPT), hydroxyurea (HU), or mitomycin C (MMC). For each condition, the individual tail moments of at least 75 cells are presented as a dot plot; the mean tail moment is denoted by a horizontal blue line, and the SEM is indicated by error bars. *p* values as determined by the paired Student’s *t* test are non-significant (ns), <0.001 (***), or <0.0001 (****). (**b**) Chromosomal abnormalities were evaluated in isogenic *Ctip*^T855A/T855A^ (clone 8) and Ctip^+/+^ (clone 7) MEFs. Cells were left untreated (no) or exposed to either mitomycin C (MMC) or camptothecin (CPT), and the numbers of chromosome aberrations (fusion, chromatid-type, and chromosome-type) per metaphase spread were determined by telomere fluorescent in situ hybridization (T-FISH). (**c**) Representative T-FISH images of individual metaphase spreads, showing DAPI-stained chromosomes (blue) and Cy3/DAPI-stained telomeres (pink).

To determine whether DNA damage is increased by loss of T855 phosphorylation, we examined cells cultured in the presence or absence of genotoxic stress using the alkaline comet assay, which detects a range of DNA lesions that include single-strand and double-strand breaks. As quantified by measuring comet tail moments, untreated C*tip*^T855A/T855A^ MEFs displayed low levels of spontaneous DNA damage that were indistinguishable from those of C*tip*^+/+^ cells (Figure 2a). Higher levels of damage were observed upon exposure to camptothecin or hydroxyurea, but again, these elevated levels were statistically indistinguishable between wild-type and mutant cells. In contrast, ionizing radiation and mitomycin C each induced significantly greater DNA damage in C*tip*^T855A/T855A^ MEFs than wild-type controls (Figure 2a), consistent with the heightened sensitivity of mutant cells to these agents (Figure 1b,c).

To ascertain whether T855 phosphorylation also preserves chromosome integrity in the face of genotoxic stress, we used the telomere-FISH assay to detect chromosomal abnormalities that arise in response to DNA damaging agents that are more (i.e., mitomycin C, Figure 1c) or less (camptothecin, Figure S3e) toxic to C*tip*^T855A/T855A^ cells. As shown in Figure 2b, untreated cells displayed few chromosome defects, and these arose at comparable levels in both C*tip*^+/+^ and C*tip*^T855A/T855A^ cells. However, mitomycin C induced a range of chromosomal aberrations, especially chromatid-like defects, that were markedly more common in C*tip*^T855A/T855A^ cells than wild-type cells. In contrast, camptothecin treatment elicited lower levels of chromosome abnormalities, and these were only modestly higher in mutant MEFs relative to wild-type cells. Thus, cells lacking the T855 phosphorylation site display increased levels of cellular hypersensitivity, DNA damage, and chromosomal rearrangements to a specific subset of genotoxic agents.

### 3.4. Homozygous Ctip^T855A/T855A^ Cells Are Impaired for Homology-Directed Repair of DNA Double-Strand Breaks

**Figure 3:**
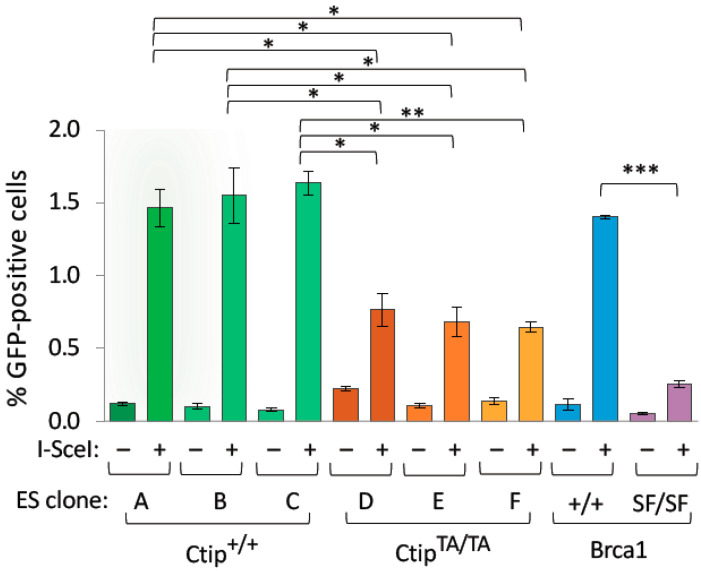
Homology-directed repair (HDR) of double-strand DNA breaks is impaired in *Ctip*^T855A/T855A^ cells. HDR efficiency was measured in independent subclones of *Ctip*^+/+^ (A, B, and C) and *Ctip*^T855A/T855A^ (D, E, and F) embryonic stem (ES) cells containing an integrated DR-GFP reporter. Cells were transfected with an empty (–) or I-*Sce*I-expressing (I-*Sce*I) vector, and the percentage of GFP-positive cells was quantified by flow cytometry. For comparison, isogenic ES cell subclones with (Brca1^SF/SF^) or without (Brca1^+/+^) a pathogenic mutation (S1598F) of the Brca1 tumor suppressor were evaluated in parallel. Each ES cell subclone was analyzed in triplicate, and error bars represent the SEM. Significant *p* values as determined by the paired Student’s *t* test are <0.05 (*), <0.01 (**), or <0.001 (***).

Previous studies have shown that DNA resection is impaired by the loss of ATR/ATM phosphorylation at the corresponding residues of human (T859) and frog (T855) CtIP [17,31]. Since resection is required for homology-directed repair (HDR) of DNA double-strand breaks (DSBs), we examined HDR function by incorporating the DR-GFP recombination reporter into a common genomic site of both wild-type (*Ctip*^+/+^) and mutant (*Ctip*^T855A/T855A^) ES cells. DSBs within the integrated DR-GFP reporter were then induced by transfecting cells with a vector encoding the I-SceI meganuclease, and HDR activity was measured by flow cytometry [39]. As shown in Figure 3, HDR levels were significantly reduced in all independent *Ctip*^T855A/T855A^ ES subclones relative to their isogenic wild-type controls. Nonetheless, *Ctip*^T855A/T855A^ cells did appear to retain some HDR activity, especially when compared to ES cells bearing a pathogenic mutation in the Brca1 tumor suppressor (*Brca1*^S1598F/S1598F^) [40]. Thus, loss of T855 phosphorylation results in a partial defect of HDR function.

### 3.5. Genotoxin-Induced DNA Resection Is Compromised in Homozygous Ctip^T855A/T855A^ Cells

**Figure 4:**
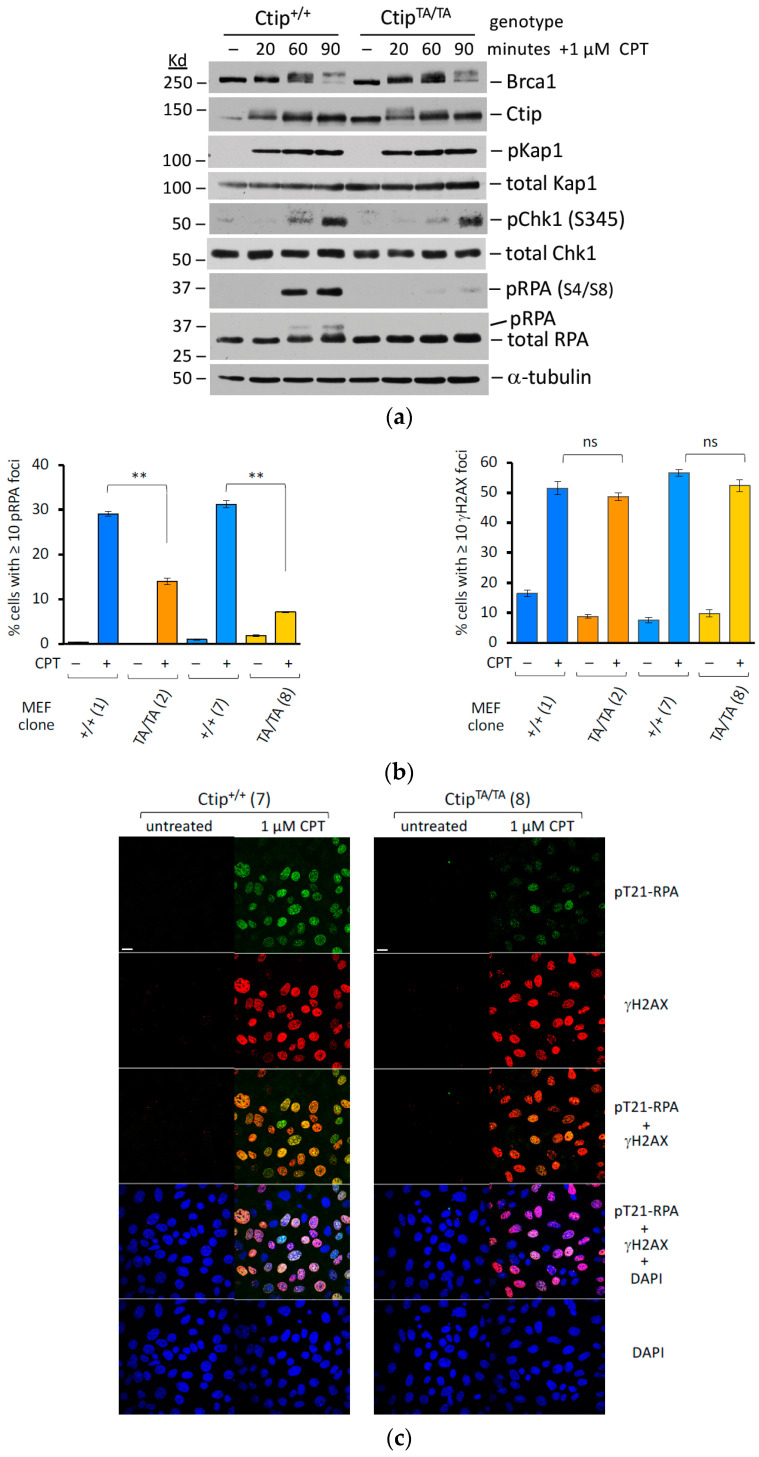
The formation of ssDNA/RPA filaments in response to DNA damage is impaired in *Ctip*^T855A/T855A^ cells. (**a**) *Ctip*^+/+^ (clone 7) and *Ctip*^T855A/T855A^ (clone 8) MEFs were exposed to camptothecin (CPT) for various time intervals (20, 60, and 90 min), and their cell lysates were immunoblotted with the indicated antibodies. (**b**) Homozygous mutant *Ctip*^T855A/T855A^ cells are defective in the formation of genotoxin-induced phospho-RPA2 foci. *Ctip*^+/+^ and *Ctip*^T855A/T855A^ MEFs, cultured with or without 1 mM camptothecin (CPT) for one hour, were evaluated by immunofluorescent microscopy for the presence of γH2AX-staining nuclear foci (left; indicative of DSBs) and phospho-RPA2-staining foci (right; indicative of ssDNA/RPA filaments). The histograms show the percentage of cells harboring >10 foci. For each culture, the Ctip genotype is indicated, and the specific MEF clone analyzed is denoted by the number in parentheses as listed in Table S1. The *p* values obtained using the paired Student’s *t* test were either non-specific (ns) or <0.01 (**). (**c**) Representative confocal images of isogenic *Ctip*^T855A/T855A^ (clone 8) and *Ctip*^+/+^ (clone 7) MEFs stained with DAPI (blue) and/or immunostained for phospho-RPA (T21) (green) and/or γH2AX (red). The white scale bars in the upper left images represent 20 µm.

To evaluate the effect of the T855A mutation on DNA resection more directly, we next examined RPA2 hyperphosphorylation in cells treated with camptothecin (CPT), an inhibitor of topoisomerase I that induces DSBs that are typically resolved by HDR. When nascent DSBs are processed by DNA resection, the resultant 3′-ssDNA overhangs are coated by the trimeric RPA complex to form ssDNA/RPA filaments, which can mediate the invasion step of HDR. Since ATM and DNA protein kinase (DNA-PK) specifically phosphorylate RPA2 subunits that are assembled within ssDNA/RPA filaments, phosphorylation of relevant RPA2 residues (e.g., S4, S8, and T21) serves as a marker of ssDNA/RPA filament formation that can be detected both biochemically and cytologically. To monitor ssDNA/RPA assembly during the DNA damage response, we compared wild-type (*Ctip*^+/+^) and homozygous-mutant (*Ctip*^T855A/T855A^) MEFs at various timepoints (20, 60, and 90 min) after CPT exposure (Figure 4a). As expected, phosphorylation of the ATM substrate Kap1 was already maximal at the earliest timepoint (20 min), while phosphorylation of the ATR substrate Chk1 developed at subsequent stages (60 and 90 min) of the CPT response. Consistent with previous work [43], DNA-PK-dependent phosphorylation of RPA2 was also observed in wild-type cells at these later stages of CPT exposure. Strikingly, however, CPT-induced RPA2 phosphorylation was ablated in *Ctip*^T855A/T855A^ cells, indicating that Ctip-T855 phosphorylation is required for DNA resection and consequent assembly of ssDNA/RPA filaments.

We also examined isogenic pairs of C*tip*^+/+^ and C*tip*^T855A/T855A^ MEF lines by immunofluorescence microscopy with phospho-specific RPA2 antibodies (Figure 4, panels b and c). Although CPT treatment induced the formation of phospho-RPA2 nuclear foci in control (*Ctip*^T855A/+^ and *Ctip*^+/+^) MEFs, focus formation was markedly reduced in *Ctip*^T855A/T855A^ cells (Figure 4b, left). In contrast, γH2AX-staining nuclear foci, which serve as a measure of DSB formation, were generated to a comparable extent by CPT treatment of both wild-type (*Ctip*^+/+^) and homozygous-mutant (*Ctip*^T855A/T855A^) MEFs (Figure 4c, right). These results indicate that CPT readily induces DSBs in both wild-type and mutant cells, but that *Ctip*^T855A/T855A^ cells fail to process these DSBs into the resected ssDNA/RPA filaments required for HDR.

### 3.6. Genotoxin-Induced DNA Resection Is Dependent on Phosphorylation of Ctip-T855, but Not the Phospho-Dependent Interaction between Ctip and Brca1

**Figure 5:**
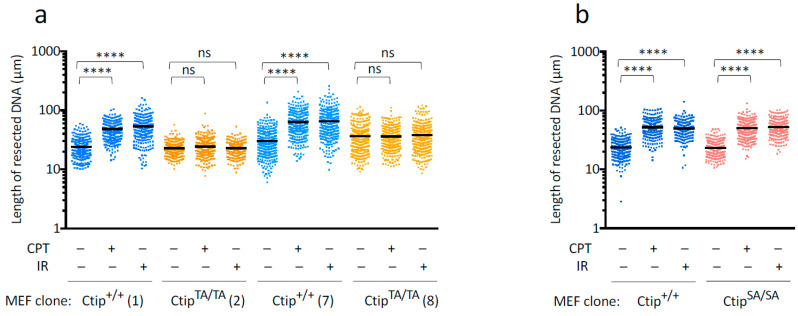
SMART analysis of DNA resection in *Ctip*^+/+^, *Ctip*^T855A/T855A^, and *Ctip*^S326A/S326A^ MEFs. (**a**) DNA fibers were prepared from pre-labeled (10 μM BrdU for 24 h) cell cultures that were left untreated (–) or exposed to either ionizing radiation (IR) or camptothecin (CPT). For each condition, the track lengths of at least 300 individual DNA fibers are presented as a dot plot, and the average track length is denoted by a horizontal bar. For each culture, the Ctip genotype is indicated, and the specific MEF clone analyzed (Table S1) is denoted in parentheses. The *p* values obtained using the Mann-Whitney rank sum test were either non-specific (ns) or <0.0001 (****). (**b**) SMART analysis of DNA resection in isogenic MEF clones expressing either wild-type Ctip (*Ctip*^+/+^) or a mutant (*Ctip*^S326A/S326A^) impaired for interaction with Brca1 [38].

To assess the effect of the T855A mutation on the extent of DNA resection, we next employed the “single-molecule analysis of resection tracks” (SMART) technique [42], which allows resection lengths to be measured on individual DNA fibers. After pre-labeling the genomic DNAs of isogenic *Ctip*^+/+^ and *Ctip*^T855A/T855A^ MEFs with 10 μM BrdU for 24 h, parallel cultures were then (1) left untreated, (2) exposed to 10 Gy of ionizing radiation and cultured for an additional hour, or (3) cultured for an additional hour in the presence of 10 μM CPT. The genomic DNAs were then extracted, stretched on glass slides, stained with antibodies that recognize incorporated BrdU nucleotides within ssDNA (exposed by resection) but not within dsDNA, and individual DNA fibers were visualized by immunofluorescence microscopy. As shown in Figure 5a, the average lengths of the resected DNA strands in *Ctip*^+/+^ cells were markedly increased upon exposure to either IR or CPT. Consistent with previous work with human cells [42], these genotoxins also increased resection to a similar degree in *Ctip*^S326A/S326A^ MEFs (Figure 5b), which harbor a missense mutation that ablates the interaction between Ctip and Brca1 [38]. In contrast, however, the resection lengths of *Ctip*^T855A/T855A^ cells were largely unaffected by treatment with either IR or CPT (Figure 5a). Together, these results indicate that ATR/ATM-mediated phosphorylation of Ctip-T855 is essential for the induction of DNA resection, specifically in response to genotoxic stress.

## 4. Discussion

CtIP^Sae2^ promotes efficient resection of DSB ends through multiple mechanisms [6,7]. First, by allosterically activating the MRN endonuclease, CtIP^Sae2^ triggers the “clipping” reaction that initiates short-range DNA resection. Second, CtIP^Sae2^ facilitates subsequent long-range resection by recruiting and activating addition factors, such as Exo1 and the DNA2/BLM complex. The 3′-single-strand DNA (ssDNA) overhang generated by CtIP-mediated resection can then serve as a platform for the assembly of Rad51/ssDNA nucleofilaments, a critical step in the DNA damage response required for both ATR-dependent checkpoint signaling and homology-directed repair (HDR) of DNA breaks.

Given the central role of CtIP^Sae2^ in the DNA damage response, the evolutionary conservation of its primary amino acid sequence seems rather modest. For example, recognizable homology between mammalian CtIP and yeast Sae2 is largely restricted to their C-terminal sequences, and within this “Sae2-like” domain, only a few amino acids are well conserved across the phylogenetic spectrum [4]. Of these, the most striking are two residues that serve as substrates for CDK-like and PI3KK-like kinases, respectively. Early studies showed that phosphorylation of the CDK site (T847 in human CtIP; Figure S1) promotes DNA resection [29,30] and subsequent work uncovered a specific role for CtIP-T847 phosphorylation in the MRN-mediated clipping reaction that initiates short-range resection [12,13,14]. The other highly conserved phosphorylation site is a substrate for DNA damage-inducible members of the phosphatidylinositol 3-kinase-related kinase (PI3KK) family, including the mammalian ATR and ATM proteins and their yeast orthologs Mec1 and Tel1 [6]. Although mutations of this residue, such as human CtIP-S859A and *S. cerevisiae* Sae2-T279A, severely impair genotoxin-induced DNA resection in vivo [29,30], the precise molecular mechanisms by which phosphorylation of this site promotes resection have not yet been elucidated. Of note, the ability of yeast Sae2 to trigger the clipping reaction in vitro is abrogated by mutation of its CDK phosphorylation site (S267A), but not its PI3KK site (T279A) [33]. In vitro studies with human components have shown that mutation of the PI3KK phosphorylation site (T859A) does reduce the ability of wild-type CtIP to stimulate the clipping reaction (from 43-fold to 15-fold), but to a significantly lesser degree than mutation of the CDK site (from 43-fold to 3-fold) [44]. Nonetheless, the precise molecular mechanisms by which PI3KK phosphorylation of CtIP at this site promotes DNA resection remain unclear.

Consistent with studies of human CtIP [17,31], we observed that mouse cells lacking Ctip-T855 phosphorylation (*Ctip*^T855A/T855A^) are severely impaired for both genotoxin-induced DNA resection and homology-dependent repair (HDR) of DSBs. Nonetheless, in the absence of acute stress, *Ctip*^T855A/T855A^ mice are largely indistinguishable from their wild-type and heterozygous (*Ctip*^T855A/+^) littermates in terms of viability, growth, fertility, and longevity. The modest phenotype of *Ctip*^T855A/T855A^ mice contrasts with that of Ctip-null mice, which invariably die during early embryonic development [7,23,24]. Interestingly, the viability of Ctip-null mice can be restored by ectopic expression of human CtIP, but not by a mutant lacking the CDK site (T847A) [34]. Thus, although phosphorylation of the CDK site and the ATR/ATM site are both necessary for efficient CtIP-mediated resection in response to genotoxic stress, only the former is required for normal animal development. Indeed, a similar phenomenon is observed within the B lymphocyte lineage, where B cell proliferation is dependent on phosphorylation of Ctip at its CDK site but not its PI3KK site [25,34].

At present, we do not know why Ctip^T855A/T855A^ cells are more sensitive to some (e.g., IR) but not other (e.g., CPT) forms of genotoxic stress. For example, given that the CPT-induced one-ended DSBs that arise during DNA replication are normally repaired by HDR, a process dependent on CtIP-mediated resection, it seemed surprising that these cells are relatively resistant to CPT treatment. Conversely, since IR-induced two-ended DSBs are largely repaired by canonical NHEJ, a process that is independent of CtIP-mediated resection, the IR hypersensitivity of Ctip^T855A/T855A^ was also unexpected. Conceivably, these results might reflect differing genotoxic sensitivities at certain stages of cell cycle progression. The elevated expression of CtIP protein in the S and G2 phases of cell cycle progression [41], together with the CDK-dependent phosphorylation of CtIP-T847 [29,30], likely ensure that DNA resection is robust during the cell cycle stages in which HDR occurs. Nonetheless, CtIP-mediated resection has also been observed in G1 cells, where CDK activity is limited [45,46,47,48]. Indeed, when G1 cells are exposed to ionizing radiation, CtIP-T847 can instead be phosphorylated by the Plk3 kinase, allowing a resection-dependent mode of non-homologous end-joining (NHEJ) to repair a subset of DSBs with slower kinetics than canonical NHEJ [45,46]. However, since CtIP protein levels and CDK activity are both markedly lower in G1 cells, the DNA resection required for this mode of repair may be more dependent on genotoxin-induced Ctip-T855 phosphorylation during the G1 phase than during the S phase. If so, then this dependency might explain why Ctip^T855A/T855A^ cells display sensitivity to IR but not CPT exposure. At the same time, we cannot dismiss the possibility that the observed pattern of genotoxin sensitivity is determined by as yet undefined resection-independent functions of Ctip-T855 phosphorylation.

Despite the relatively normal phenotype of unstressed *Ctip*^T855A/T855A^ mice, their hypersensitivity to ionizing radiation (Figure 1a) implies that phosphorylation of Ctip-T855 does have physiological relevance when animals face environmental challenges that elicit certain forms of DNA damage. Interestingly, Ctip-T855 phosphorylation is also required in biological settings where the NHEJ pathway of DSB repair is compromised, such as for the survival of Xrcc4/Tp53-deficient mice, cytokine activation of Xrcc4/Tp53-deficient B cells, and lymphomagenesis in *DNA-PKcs*^−/−^*Tp53*^−/−^ mice [11,35]. Thus, Ctip-T855 phosphorylation appears to be relevant whether DNA damage is elicited exogenously, by genotoxin exposure, or endogenously due to persistent DSB repair defects. Taken together, these observations suggest that phosphorylation of the PI3KK site serves to enhance resection in response to acute genotoxic stress, while phosphorylation of the CDK site—and thus induction of the MRN-mediated clipping reaction—is essential for cell viability and animal development even in the absence of stress.

In addition to its central role in DNA resection, CtIP has also been implicated in other cellular processes, such as transcriptional co-repression, cell cycle regulation, and chromosome segregation [7]. Nonetheless, the embryonic lethality of mice expressing Ctip lacking the conserved CDK site (T847A) [34] suggests that DNA resection—and more particularly, the clipping reaction—may be the CtIP function most important for normal animal development. Given that DNA resection is severely abrogated in vivo by loss of either the CDK site (T847) or the neighboring PI3KK site (T859), it is surprising that mice lacking the PI3KK site display such a modest phenotype. Elucidating the molecular mechanisms by which phosphorylation of the conserved PI3KK site promotes resection may help to resolve this enigma.

## Data Availability

The original data presented in this study are included in the article; further inquiries can be directed to the corresponding author.

## Supplementary Materials

The following supporting information can be downloaded at: https://www.mdpi.com/article/10.3390/cells12232762/s1, Figure S1. Conserved features of the CtIP/Sae2 polypeptides; Figure S2. Design of the *Ctip*^T855A^ allele; Figure S3. Ctip protein expression and genotoxin sensitivities of MEF clones bearing wildtype and/or mutant *Ctip* alleles; and Table S1. The isogenic mouse embryonic fibroblast (MEF) lines.
