# Supplementary material for: ATM/ATR Phosphorylation of CtIP on Its Conserved Sae2-like Domain Is Required for Genotoxin-Induced DNA Resection but Dispensable for Animal Development
Source: Cells. 2023 Dec 4;12(23):2762. doi: 10.3390/cells12232762 (PMC10706839; doi:10.3390/cells12232762)
Supplement: Supplementary file 1 [file cells-12-02762-s001.zip › cells-2653686-SM 1.pdf]

Supplementary Figure legends

Supplementary Figure S1. Conserved features of the CtIP/Sae2 polypeptides.
The C-terminal sequences of *S. cerevisiae* Sae2 exhibit amino acid homology with the corresponding regions of vertebrate CtIP proteins, such as human CtIP, mouse Ctip, and Xenopus xCtIP. This conserved "Sae2-like domain" includes both a CDK-like (T847 in humans) and a PI3KK-like (T859 in humans) phosphorylation site. The vertebrate CtIP orthologs also share an N-terminal coiled-coil domain that mediates CtIP homodimerization, a phosphorylation site that facilitates BRCA1 binding (S327 in humans), and an interaction site for the CtBP transcriptional co-repressor (PLDLS).

Supplementary Figure S2. Design of the *Ctip*^{T855A} allele.
A map of the wildtype mouse *Ctip* locus encompassing exons 16-19 is shown (**a**), followed by diagrams of the targeting construct (**b**), the *Ctip*$^{T855A\text{-}neo}$ allele generated upon homologous recombination (**c**), and the *Ctip*^{T855A} allele generated by Cre-mediated recombination (**d**). For the targeting construct, the T855A mutation was introduced into exon 18 and a neomycin expression cassette (neo) flanked by *loxP* recombination signals (pink triangles) was inserted into the *BstXI* site of intron 18. An HSV thymidine kinase (HSV-TK) gene cassette was included in the targeting vector for negative selection. Restriction sites are: HindIII (H3), EcoRV (RV), BglII (B2), BstXI, XhoI, Sac1, PmeI, NotI, and BamHI (Bam). The 5′ and 3′ *Ctip* probes used for Southern analysis are also shown. An example is provided of a nucleotide sequence histogram obtained from DNA of a homozygous *Ctip*$^{T855A/T855A}$ MEF clone (**e**), along with an illustration of *Ctip* genomic sequences encompassing exon 18 and the mutant T855A codon (highlighted in a red box) of the *Ctip*^{T855A} allele. The PCR primers (Ctip-19 and Ctip-O) used for nucleotide sequence analysis are also depicted.

Supplementary Figure S3. Ctip protein expression and genotoxin sensitivities of MEF clones bearing wildtype and/or mutant *Ctip* alleles.
(a) Heterozygous *Ctip*$^{T855A/+}$ mice were intercrossed, and mouse embryonic fibroblasts (MEFs) were derived from the day E13.5 embryos of three matings (Table S1). MEFs were cultured in the presence or absence of camptothecin (1μM CPT for 1 hour) and Ctip protein expression was examined by immunoblotting with a Ctip-specific antiserum. For each culture, the *Ctip* genotype is indicated and the specific MEF clone analyzed is denoted by the number in parentheses as listed in Table S1.
(b-f) Colony survival analysis of isogenic clones of *Ctip*$^{+/+}$, *Ctip*$^{T855A/+}$ and *Ctip*$^{T855A/T855A}$ mouse embryonic fibroblasts (MEFs) exposed to olaparib **(b)**, ultraviolet light (UV) **(c)**, hydroxyurea (HU) **(d)**, neocarzinostatin (NCS) **(e)**, or etoposide **(f)**. Survival was quantified as the percentage of colonies on genotoxin-treated cultures relative to untreated cultures. Each condition was tested in triplicate, and error bars represent SEM. For each culture, the *Ctip* genotype is indicated and the specific MEF clone analyzed (Table S1) is denoted in parentheses.

Supplementary Table

Supplementary Table S1. The isogenic mouse embryonic fibroblast (MEF) lines used in this study were derived from the day E13.5 progeny of three *Ctip*$^{T855A/+}$ intercrosses.

MEF line	Intercross	Genotype (embryo #)
1	0304M1	*Ctip*$^{+/+}$ (E6)
2	0304M1	*Ctip*$^{TA/TA}$(E10)
3	0304M1	*Ctip*$^{+/+}$ (E5)
4	0304M1	*Ctip*$^{TA/TA}$(E1)
5	0304M1	*Ctip*$^{TA/+}$ (E2)
6	0304M1	*Ctip*$^{TA/+}$ (E8)
7	0311M1M2	*Ctip*$^{+/+}$ (E6)
8	0311M1M2	*Ctip*$^{TA/TA}$(E5)
9	0311M1M2	*Ctip*$^{+/+}$ (E1)
10	0311M1M2	*Ctip*$^{TA/+}$ (E3)
11	211M1	*Ctip*$^{+/+}$ (E3)
12	211M1	*Ctip*$^{TA/TA}$(E5)
13	211M1	*Ctip*$^{TA/+}$ (E2)
14	211M1	*Ctip*$^{TA/+}$ (E1)
