# Supplementary material for: ATM/ATR Phosphorylation of CtIP on Its Conserved Sae2-like Domain Is Required for Genotoxin-Induced DNA Resection but Dispensable for Animal Development
Source: Cells. 2023 Dec 4;12(23):2762. doi: 10.3390/cells12232762 (PMC10706839; doi:10.3390/cells12232762)
Supplement: Supplementary file 1 [file cells-12-02762-s001.zip › cells-2653686-supplementary.pdf]

Figure S1

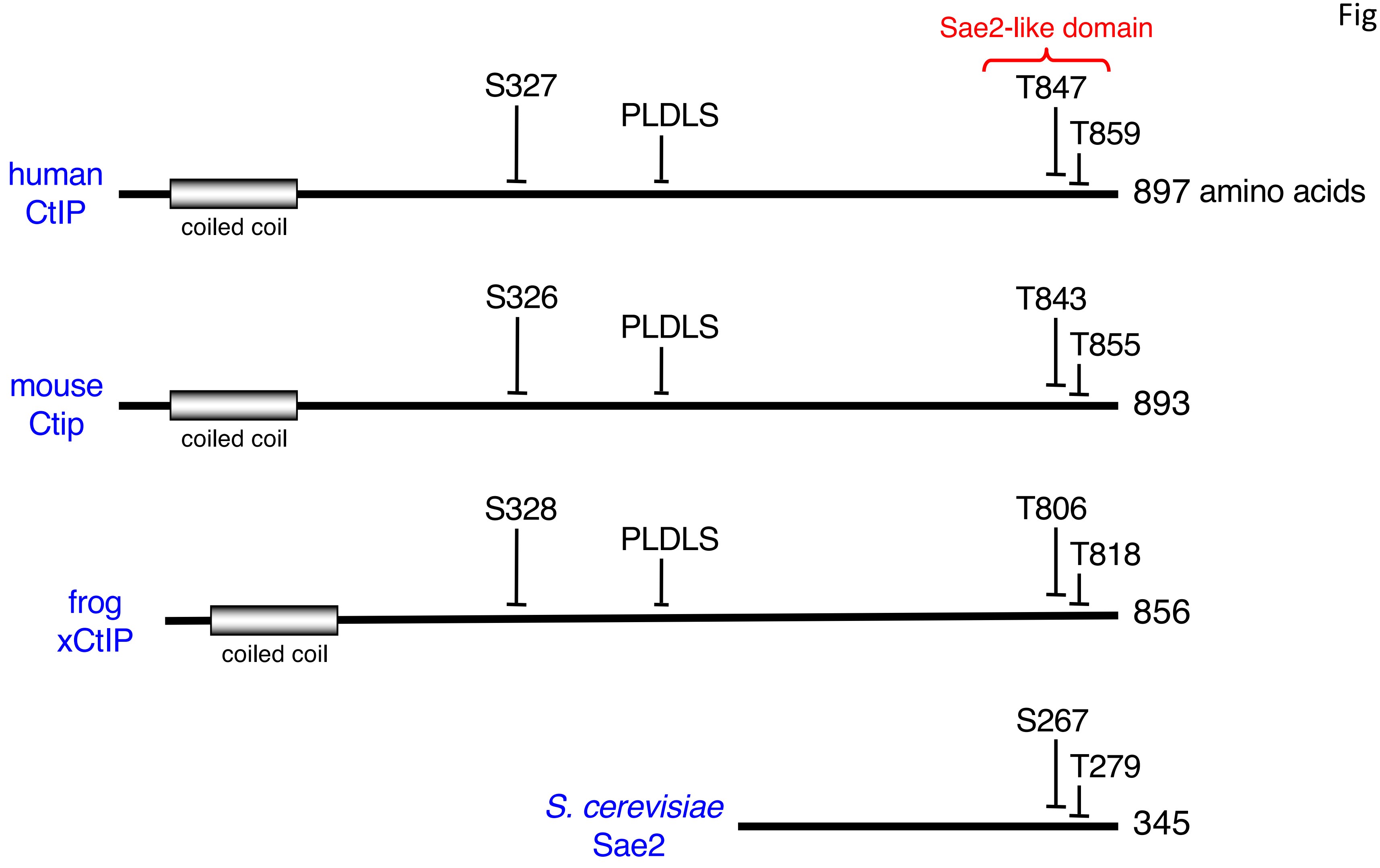

Sae2-like domain
S327
PLDLS
T847
T859
human CtIP
coiled coil
897 amino acids
S326
PLDLS
T843
T855
mouse Ctip
coiled coil
893
S328
PLDLS
T806
T818
frog xCtIP
coiled coil
856
S267
T279
S. cerevisiae Sae2
345

Figure S2a-d

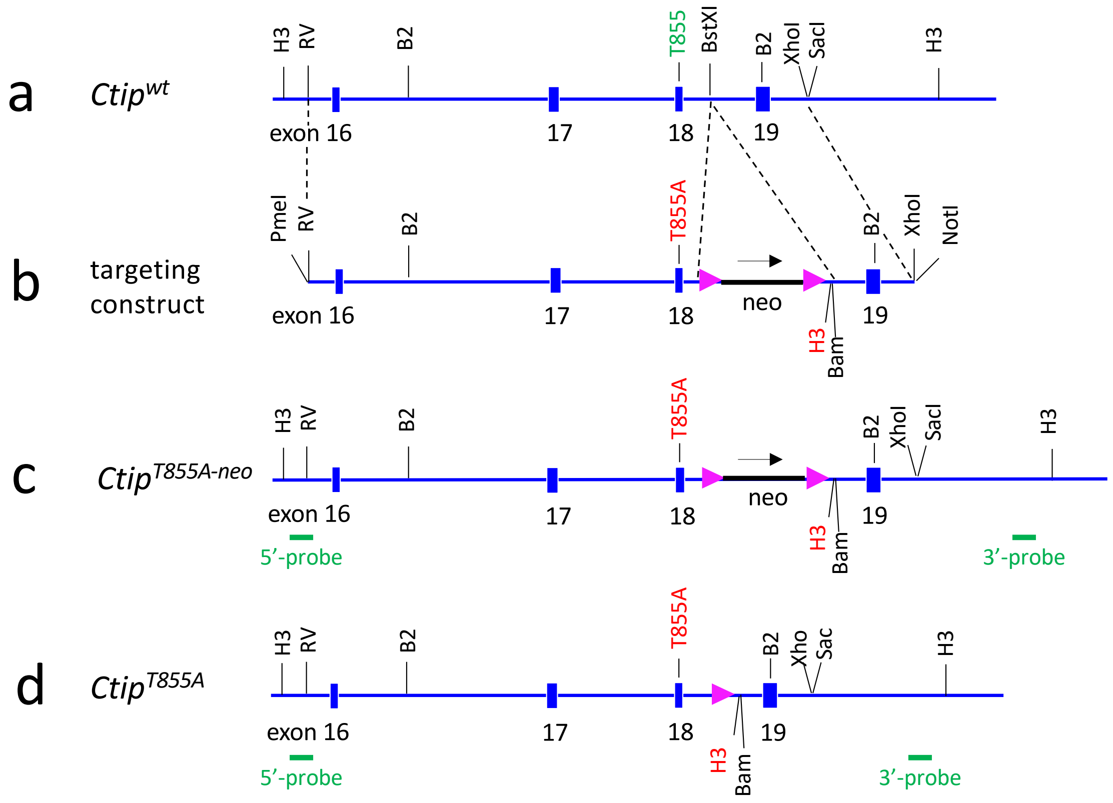

a
Ctipwt
H3
RV
B2
T855
BstXI
B2
XhoI
SacI
H3
exon 16
17
18
19
b
targeting construct
PmeI
RV
B2
T855A
B2
XhoI
NotI
exon 16
17
18
neo
19
H3
Bam
c
CtipT855A-neo
H3
RV
B2
T855A
B2
XhoI
SacI
H3
exon 16
17
18
neo
19
H3
Bam
5'-probe
3'-probe
d
CtipT855A
H3
RV
B2
T855A
B2
Xho
Sac
H3
exon 16
17
18
19
H3
Bam
5'-probe
3'-probe

Figure S2e

Point mutant validation of Ctip-T885A/T855A Mefs:

**File: 0304M1-6-mctip-19.ab1**

Geospiza
www.geospiza.com

Sample Name: 0304M1-6-mctip-19
Mobility: KB_3730_POP7_BDTv3.mob
Spacing: 15.168
Comment: 1599705-A2-Wu-Baer, Foon-10-280656606-FW09

Signal Strengths: A = 3088, C = 2253, G = 2978, T = 3631
Lane/Cap#: 16
Matrix: n/a
Direction: Native

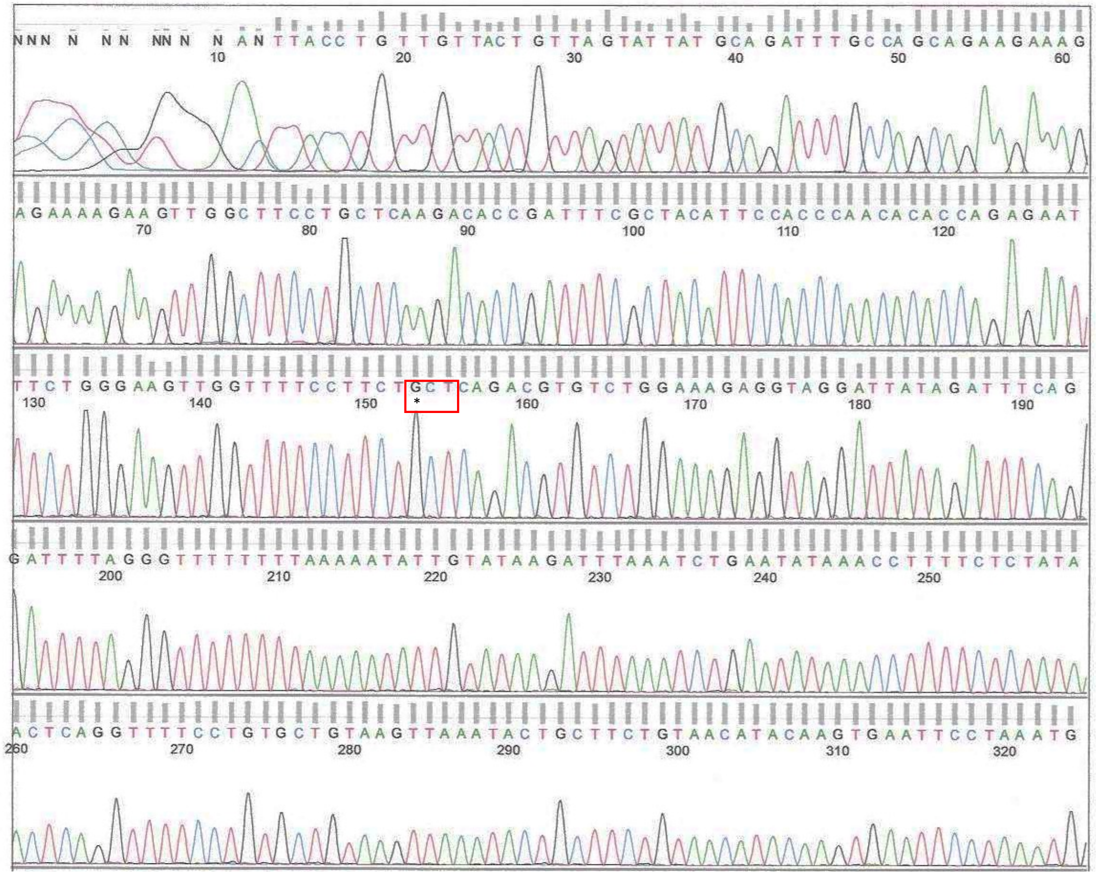

Figure S2f

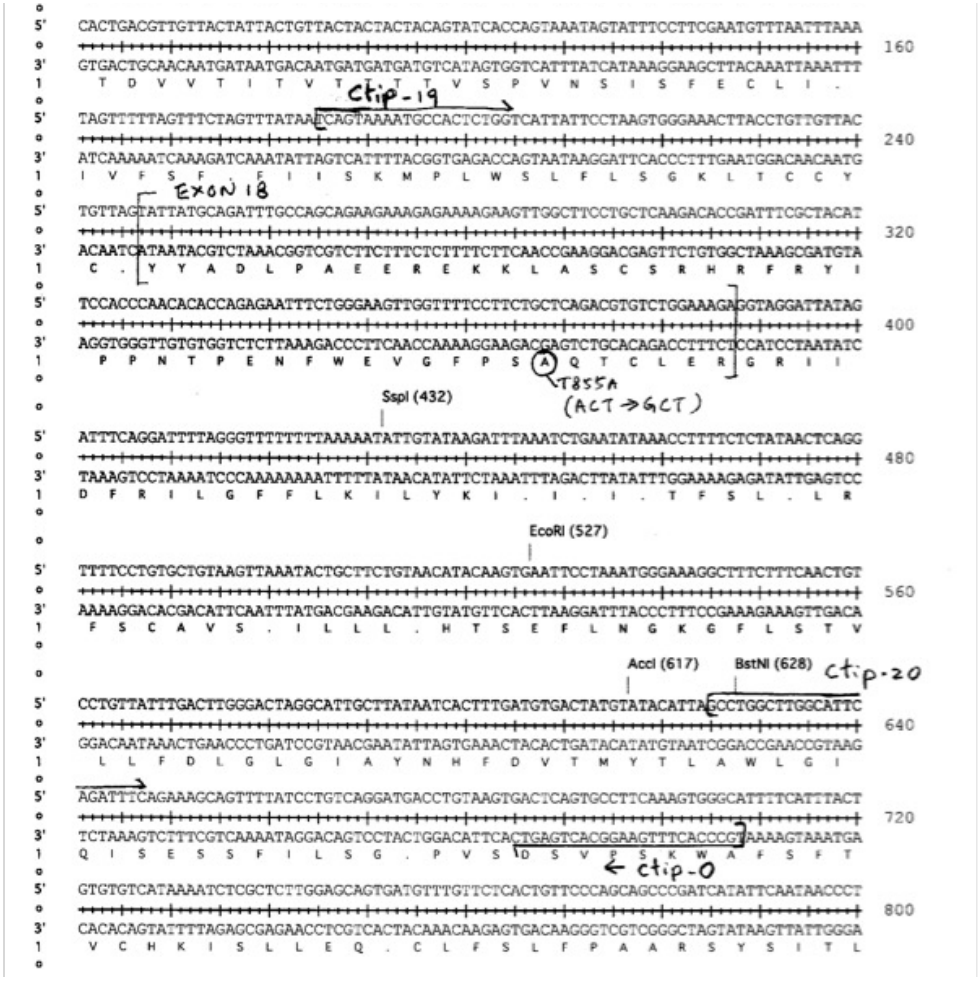
Ctip-19
EXON 18
T855A
(ACT→GCT)
SspI (432)
EcoRI (527)
AccI (617)
BstNI (628)
Ctip-20
← Ctip-O
160
240
320
400
480
560
640
720
800

Figure S3a

a

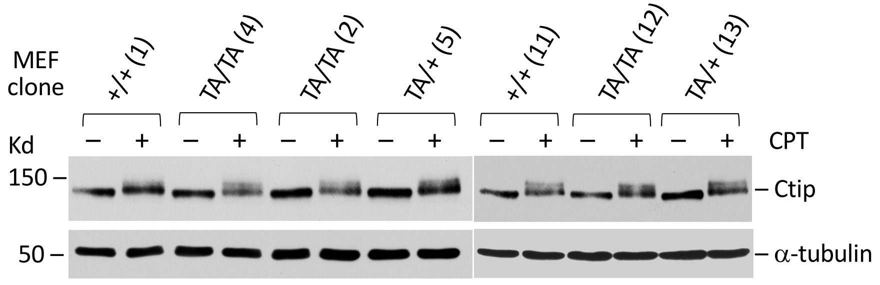
MEF clone
+/+ (1)
TA/TA (4)
TA/TA (2)
TA/+ (5)
+/+ (11)
TA/TA (12)
TA/+ (13)
Kd
– + – + – + – + – + – + – +
CPT
150
Ctip
50
α-tubulin

Figure 3b-e

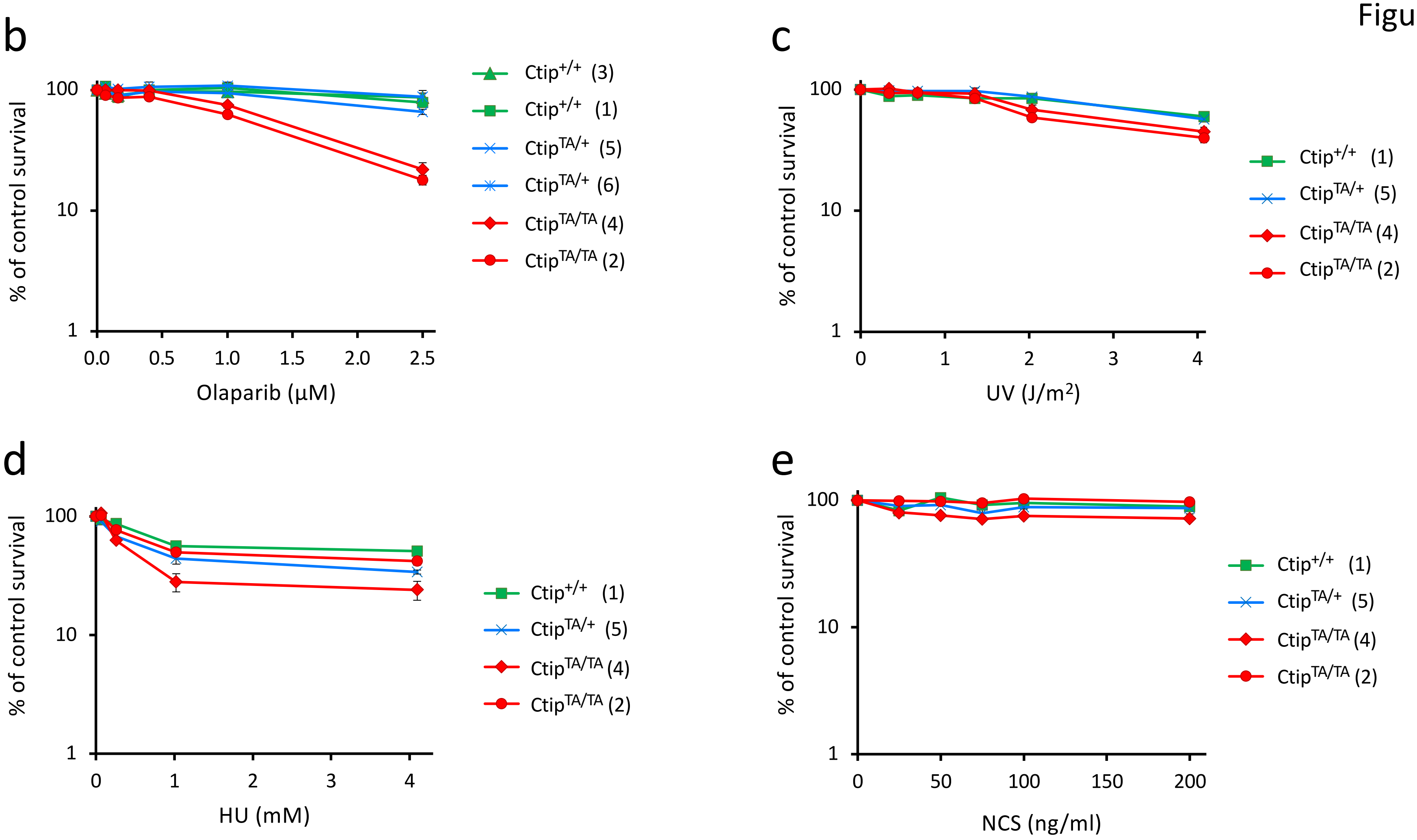

b
% of control survival
100
10
1
0.0
0.5
1.0
1.5
2.0
2.5
Olaparib (μM)
Ctip$^{+/+}$ (3)
Ctip$^{+/+}$ (1)
Ctip$^{TA/+}$ (5)
Ctip$^{TA/+}$ (6)
Ctip$^{TA/TA}$ (4)
Ctip$^{TA/TA}$ (2)
c
% of control survival
100
10
1
0
1
2
3
4
UV (J/m$^2$)
Ctip$^{+/+}$ (1)
Ctip$^{TA/+}$ (5)
Ctip$^{TA/TA}$ (4)
Ctip$^{TA/TA}$ (2)
d
% of control survival
100
10
1
0
1
2
3
4
HU (mM)
Ctip$^{+/+}$ (1)
Ctip$^{TA/+}$ (5)
Ctip$^{TA/TA}$ (4)
Ctip$^{TA/TA}$ (2)
e
% of control survival
100
10
1
0
50
100
150
200
NCS (ng/ml)
Ctip$^{+/+}$ (1)
Ctip$^{TA/+}$ (5)
Ctip$^{TA/TA}$ (4)
Ctip$^{TA/TA}$ (2)

Figure S3f

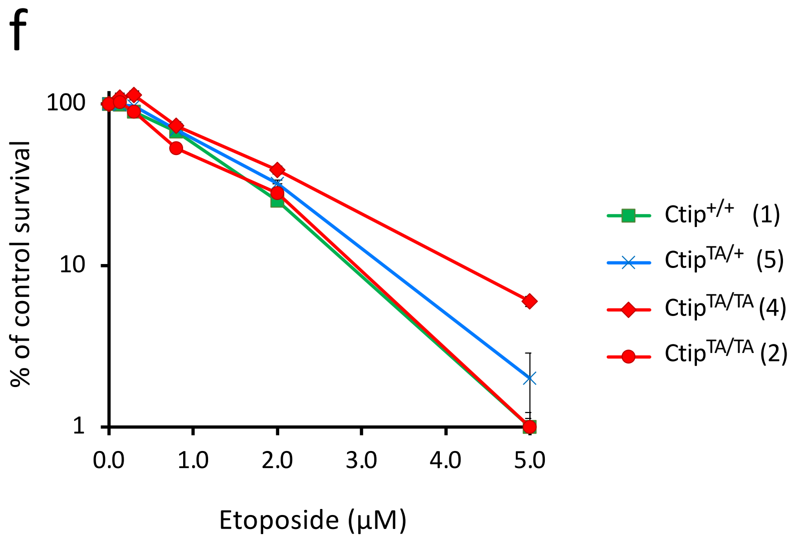

f
100
10
1
% of control survival
0.0
1.0
2.0
3.0
4.0
5.0
Etoposide (μM)
Ctip+/+ (1)
CtipTA/+ (5)
CtipTA/TA (4)
CtipTA/TA (2)
